# Optimizing the Cement Rheology and Hydrophobicity Using Polycarboxylate Ether (PCE)-Based Grinding Aids

**DOI:** 10.3390/polym17223002

**Published:** 2025-11-12

**Authors:** Kenan Çinku, Ebru Dengiz Özcan, Şenel Özdamar, Hasan Ergin

**Affiliations:** 1 Department of Mining Engineering, Istanbul University-Cerrahpaşa, Istanbul 34500, Türkiye; 2 Department of Mining Engineering, Istanbul Technical University, Istanbul 34469, Türkiye; 3 Department of Geological Engineering, Istanbul Technical University, Istanbul 34469, Türkiye

**Keywords:** polymer-based grinding aids (PCE), Portland cement, surface tension, water contact angle (WCA), hydrophobicity, rheology, FT-IR spectroscopy

## Abstract

Newly developed polymer-based grinding chemicals demonstrate superior dispersion, grinding, and strength outcomes compared to traditional amine-based additives. This study provides a comprehensive analysis of the mechanisms underlying the improved performance of polymers in the grinding process. It examines the influence of polymer-based grinding aids (A1-A2-A3) on the hydrophobicity and rheological behavior of CEM I 42.5 R Portland cement. A systematic analysis was conducted using six different grinding aids, comprising three synthesized polycarboxylate ether (PCE)-based polymers and three commercial amine group products. Key properties, including surface tension, hydrophobicity (water contact angle, WCA), slump flow, FT-IR, and rheological parameters, were evaluated. Among the compounds tested, the A2 polymer exhibited the most favorable performance, achieving a high contact angle (131.7°), low surface tension (56.7 dyn/cm), and enhanced mortar fluidity (25 cm slump flow). FT-IR spectroscopy confirmed strong interactions between A2 and cement particles, particularly in the CH_3_ bonding regions. Rheological analyses further revealed that A2—2.5 g significantly decreased viscosity and improved shear stress response, indicating superior dispersion and water reduction capability. The findings highlight A2 as a promising eco-efficient additive for enhancing the efficiency, performance, and workability of cementitious systems through polymer-based grinding technology.

## 1. Introduction

The use of admixtures is a crucial component in the production processes of cement and concrete. Over the past decade, there have been significant advancements in the field of admixtures, with notable developments affecting the performance of cement, concrete, and mortars. Traditionally, these admixtures were classified as amine-based plasticizers. However, a new class of superplasticizer, known as polycarboxylate ether (PCE), has been introduced. These materials are characterized by a polymethacrylic or alkyl acid backbone with side chains containing methoxy-polyethylene glycol groups [1,2]. A multitude of structural variations can be achieved through the modulation of backbone substitution levels and the variation in side-chain lengths. The aforementioned attachments are associated with a steric hindrance. This phenomenon, defined as the occurrence of adsorption on cement grains that have already undergone surface adsorption, is of particular interest in this study. The function of steric hindrance is to prevent the agglomeration of particles. The release of associated water has been demonstrated to enhance workability, with the potential to achieve water reductions of up to 40% [3]. These admixtures are classified as high-range water-reducing agents and they can be utilized at significantly higher dosages than conventional water-reducing admixtures without causing adverse side effects, such as gross retardation of set. It can thus be concluded that a greater reduction in the volume of mixing water is possible in the case of concretes of normal workability (up to 30%) or for obtaining flowing without excessive addition of water to the mix [4].

All superplasticizers consist of high molecular weight, water-soluble polymers, the majority of which are synthetic compounds. The solubility of the substance under investigation is ensured by the presence of adequate hydroxyl, sulfonate or carboxylate groups attached to the main organic repeating unit, which is normally anionic. PCEs are characterized by a molecular weight ranging from 20,000 to 80,000 with side chain lengths being mixed and varied, thereby resulting in a range of properties. It has been demonstrated that certain PCEs are susceptible to hydrolysis in alkaline media, a process that may compromise their effectiveness [5,6]. These substances exhibit a high degree of adsorption, often forming a multilayer of chemisorption on individual grains of cement [7].

The concept of hydrophobicity entails the introduction of agents as solutions or suspensions into concrete, where they form as insoluble calcium salts. The zeta potential (ZP) refers to charge formation on the surface of the particle, which can be attributed to various mechanisms such as ionization, surface group dissociation, or ion adsorption. The magnitude and sign of the charge depend on the pH of the medium and the ions present in the solution. As the ZP value increases, the repulsive force between particles also increases, thereby enhancing the stability of the suspension. Specifically, the decline in Ca^2+^ ions results in a concomitant decrease in zeta potential, directed in the negative direction. This direct decrease is inversely related to the increase in SO_4_^2^−^^ bonding [8,9]. The underlying cause of these behaviors can be attributed to the significant increase in zeta potential (up to −30 to −40 mV) induced by polymer-based chemical additives [10]. These additives form a water-repellent coating on the concrete surface and on the inner walls of capillary pores, thereby increasing the angle of contact of liquid water and thereby inhibiting its wetting capability. In exceptional circumstances, it has been hypothesized that water may be expelled from a concrete or mortar surface as opposed to being adsorbed [11].

Water-reducing admixtures (plasticizers) are hydrophilic surfactants that, when dissolved in water, deflocculates and disperse cement particles. It has been demonstrated that by preventing the formation of an agglomerate of cement particles in suspension, a reduced quantity of water is required to produce a paste of a given consistency or concrete of a particular workability. This phenomenon can be attributed to the adsorption of high molecular weight anions, which are components of these admixtures, onto cement surfaces. This results in the mutual repulsion of individual particles, thereby reducing interparticle friction [12].

**Figure 1:**
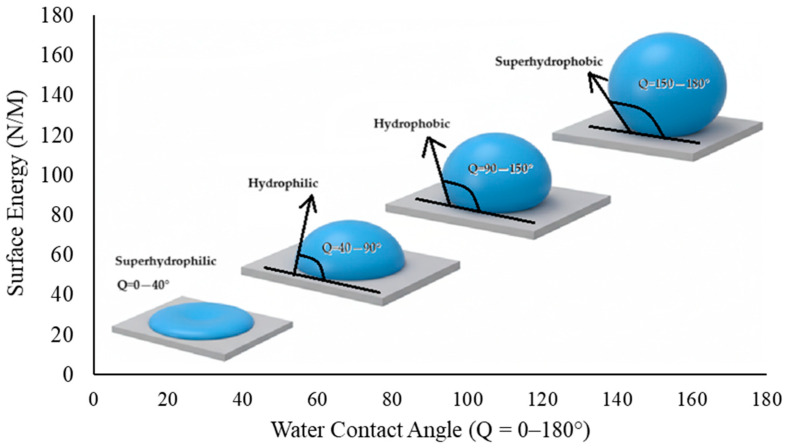
Correlation between WCA and surface energy (derived from [13]).

The propensity of the cement surface to exhibit hydrophilic or hydrophobic characteristics is dependent on the interesting balance between the cement grains and the grinding agent. As the utilization rate of the grinding chemical increases, the repulsive force between the cement grains increases and the surface’s requirement for water decreases. For a cement surface to be defined as hydrophobic, the system’s contact angle must exceed 90°. However, it has been demonstrated that increasing the rate of use of grinding chemicals renders the function of the grinding chemical reversible after a certain point. This phenomenon concomitantly results in a decrease in the water-shearing property of the grinding chemical. Consequently, the system becomes less hydrophobic. Hydrophobicity is defined as the property of a substance that causes molecules on its surface to be repelled from the bulk of an aqueous solution, and hydrophobic surfaces have low surface energy [12]. The water contact angle (WCA) test is used to determine whether a material is hydrophobic or hydrophilic. The correlation between surface energy and WCA is demonstrated in Figure 1.

A molecular simulation study of C_3_S cement showed that the main chains of PCE containing carboxylate groups and polyether side chains are adsorbed onto dry C_3_S surfaces. It has been demonstrated that the branching of the molecule prevents its complete adsorption in a flat configuration. However, linear molecules consisting of polyethylene oxide chains are capable of achieving complete flat adsorption. A specific steric effect occurs if branching makes flat adsorption impossible or if there is significant overlap of polymer chains [14]. The adsorption of PCE on C_3_S surfaces is predominantly attributable to electrostatic interactions, with Van der Waals interactions being negligible. Calcium ions of C_3_S tend to bind with PCE, with a degree of repulsion from the C_3_S surface being observed to a certain extent. The surface of C_3_S undergoes a slight reconstruction as a result of the adsorption of PCE. It is also noteworthy that silicate ions are observed to emanate from the C_3_S surface [15]. The mechanism of action can be outlined as follows: the polar sides of adsorbed PCE molecules offer partial compensation to the polar surface, thereby reducing it through the shielding effect of their non-polar alkylene groups. It can thus be concluded that the attractive force between particles decreases in proportion to decreasing surface polarity. This in turn results in a minimization of the tendency towards agglomeration formations due to steric stabilization. PCE molecules have been shown to remain largely intact during the grinding process. This is due to the water-reducing effect of PCE, which has been observed during the mixing of water and cement, as in the case of concrete [16,17,18,19].

The adsorption of polymer-based grinding aids (GAs) onto cement particles has been shown to result in steric and electrostatic effects that contribute to a reduction in both interparticle agglomeration and the water demand of cement, positively affecting its strength [20,21,22,23,24].

This research indicates that silanols are the reason why polymer-based grinding chemicals are preferred over amine groups as cement grinding chemicals in this manuscript. Silanols occupy a pivotal position within the domain of polymer systems, being formed by the combination of Si and OH ions. It has been demonstrated that the system under consideration has the capacity to be rendered either hydrophobic or hydrophilic. Accordingly, this study characterizes and examines anionic polymer-based grinding aids with respect to the surface tension, contact angle, rheology and FT-IR analyses of the cement particles.

## 2. Materials and Methods

### 2.1. Materials

The cement employed in this study was CEM I 42.5 R Portland cement (PC), manufactured by NUH Cement Factory in Hereke/Kocaeli-TÜRKİYE. CEM I 42.5 R is a high-early strength and high-final strength cement. These are obtained by grinding Portland cement clinker with gypsum as a setting regulator in accordance with EN 197-1 standards [22]. The composition of the material is 91% clinker, 5% limestone and 4% gypsum. The chemical composition of the clinker was analyzed by XRF and is given in Table 1.

#### Synthesis of PCEs

The polymers A1–A3 were synthesized at a temperature of 30 °C. The synthesis of A1–A3 was completed within 2 h, while A2 required 3 h and 40 min to synthesize. The synthesis of vinyl polyethylene glycol (VPEG) was accomplished through the utilization of acrylic acid, a copolymer, in a specific synthetic process. This is an additional reaction initiated by a free radical starting chemical. Azobisobutyronitrile (C_8_H_12_N_4_) was used as an initiator. The substance functions effectively at low temperatures and is classified as an organic solvent. The chemicals A1, A2, and A3 were synthesized from a VPEG-based polymer, and their general formulas are given in Table 2 together with those of other well-known commercial products. AMA-6E is a commercial product produced by a factory of French origin and sold in Turkey as a cement grinding chemical. Moreover, in the cement industry, both TEA and TIPA are used as grinding aids [7,11].

**Figure 2:**
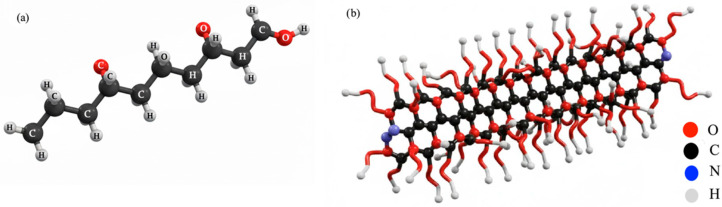
General illustrations of VPEG (**a**), A1–A2, and A3 (**b**) chemical aids.

The chemicals A1, A2 and A3 were synthesized from a VPEG-(CH_2_=CH-CH_2_-[O-CH_2_-CH_2_]n-OH)-based polymer by radical polymerization and their general illustrations are given in Figure 2.

One of the monofunctional polyglycols was vinyl ethoxy polyethylene glycol (V-PEG), serving as a reactive polymer providing hydrophilicity and steric stabilization in diverse applications. Its single hydroxyl group enables versatile chemical modifications with compounds such as carboxylic acids, isocyanates, and epoxides. V-PEG, synthesized via ethoxylation, inherently contains a polymerizable double bond, facilitating copolymerization and comb polymer formation. Furthermore, V-PEG derivatives play a significant role in organopolysiloxane synthesis through hydrosilylation.

It is evident that TEA is an amine, defined as a functional group containing a basic nitrogen atom, and an ethanol, understood as a source of hydroxyl groups. In contrast, TIPA is classified as an alcohol-amine compound, incorporating three isopropanols, a feature that differentiates it from an amine. It is evident that both substances possess low molecular weights (respectively 149.19 g/mol and 191.27 g/mol) and are classified as short-chain simple organic compounds. The presence of alcohol in their composition gives rise to the manifestation of polarity. The presence of three alcohols in TIPA, as opposed to the single alcohol in TEA, results in a higher degree of polarity. The mechanisms of action of these compounds are similar, in that they neutralize surface charges on cement particles. This prevents microcrack closure, as well as the agglomeration of particles and the coating of mill components. However, it has been reported that TIPA increases late-age strength more effectively than TEA. Being simple organic compounds, high dosages are required for these compounds to be effective as cement grinding aids (0.04–1%) [25,26,27,28].

The newly synthesized high molecular weight grinding aid under investigation in this study provides the same effect even at low dosages of up to 50%. It has been demonstrated that even at this low dosage, grinding performance is increased by 30–32%, and the amount of energy required for grinding can be reduced by up to 7–9% [7]. In the course of the synthesis stage, the vinyl double bonds in the small-molecule VPEG monomer were broken with azobis and converted into single covalent bonds, thereby forming a very high molecular weight and long-chain polymer (PCE). The primary effect of this polymer can be summarized as follows: it rapidly neutralizes surface charges on cement particles, then provides a dispersion effect by converting them into negatively surface-charged particles at the optimum dosage, thereby preventing the closure of microcracks, particle agglomeration, and coating of mill components. Furthermore, the addition of this substance to the mixture serves to reduce water demand, thereby creating a low-viscosity, self-leveling mixture [11]. The long and complex structure of the substance in question facilitates the coating of cement grains, thereby increasing the material’s hydrophobicity. It is hypothesized that an augmentation in strength may be attainable through a reduction in water demand by means of the distribution of cement particles via electrostatic repulsion and steric hindrance (i.e., spatial constraints). It is hypothesized that high hydrophobicity may also have the potential to provide positive contributions to the strength, duration and protection of steel reinforcement in concrete and mortar applications containing this grinding additive [29,30,31].

### 2.2. Methods

In order to ensure reproducibility and statistical robustness, a total of measurements was performed for each sample, encompassing both the fixed dosage group (2.5 g; six samples) and the variable A2 dosage group (seven samples). Each measurement was carried out over three consecutive cycles. To further minimize experimental bias and validate data reliability, the complete measurement protocol was repeated the following day under strictly identical conditions.

#### 2.2.1. Surface Tension of Grinding Aids

The solutions of the cement grinding chemicals (Reference, TEA, TIPA, AMA/6E, A1, A2, A3) and A2-coded PCE-based grinding aids in varying concentrations were prepared in under controlled laboratory conditions.

An initial solution of 100 mL was prepared for each polymer-based grinding chemical. For example, to prepare a 1.5 wt.% A2 solution, 1.5 g A2 was dissolved in 98.5 g deionized water (100 mL total). The samples were then subjected to a shaking process by hand for 15 min. The surface tensions of the prepared solutions (see Table 3) were measured using a goniometer (Krüss GmbH, model K6-95572, Berlin, Germany) by the DuNouy Ring Method. This method measures the force on a ring as the liquid’s lamella is withdrawn, enabling surface and interfacial tension determination. The solution compositions are provided in Table 3.

#### 2.2.2. Contact Angle of PCE’s

Thirteen pellets were created using samples of cement that had previously been ground with different grinding chemicals (TEA, TIPA, AMA/6E, A1, A2, and A3). The contact angles of these samples were measured using the DataPhysics OCA 15 EN (DataPhysics Instruments GmbH, Stuttgart, Germany) device. The samples were carefully positioned within the goniometer apparatus, and a precise drop of water was applied to the pellet sample in both right, left, up and down directions. The fine samples were dried in an oven at 105 °C for 24 h. After drying, the grain sizes of 20–30 g samples were reduced to <45 µm by using vibratory disc milling device with tungsten carbide grinding set. Milling process was carried out for 5 min at 1250 rpm. All dried and powdered samples were stored in a desiccator to protect from humidity. After gently pouring 1 g of boric acid into a cylindrical pellet mold, five grams of the ground sample were placed on top. The sample was then formed into a homogeneous, durable disc by applying 180 kN of hydraulic force in a press machine and waiting for 15 min. The pellet disc diameter was 32 mm. The instantaneous contact angle values were then recorded automatically from the computer connected to the device. The data obtained from all four directions for each sample were then averaged. The goniometer was used to record the drop images and automatically analyzes the drop shape over time. The morphology of the droplet is determined by the interplay of surface tension in the liquid and the density disparity between the liquid and the surrounding medium. On stable surfaces, the configuration of the drop and the contact angle are contingent on the free surface energy of the solid. A range of measurements were taken, including contact angle, surface tension, interfacial tension, surface free energy, wettability, liquid absorption, liquid retention, diffusion, surface cleanliness, surface heterogeneity, emulsion stability, etc.

#### 2.2.3. Rheology

The samples were prepared to measure shear stress and viscoelastic behaviors using Brookfield R/S Plus Rheometer (Brookfield Engineering Laboratories, Inc., Middleboro, MA, USA). Cement mortars are yield-stress fluids [24,32,33], i.e., material flow commences immediately as the applied stress exceeds the yield stress. The behavior of cement pastes, namely shear thinning or shear-thickening, is contingent on the mixing ratio and the applied shear rate and shear stress [34,35,36]. The test procedure was selected according to the material properties under investigation, for example, constant flow, thixotropic, or viscoelastic behavior in rotational or oscillatory modes. The constant flow properties of cement mortars are directly related to yield stress. Cement samples obtained from raw materials subjected to the grinding process with different grinding chemicals were prepared at a concentration of 15% [(15 g cement)/(85 g water)] and shear stress measurements were made using the rheometer.

#### 2.2.4. FT-IR Analyses

Fourier Transform Infrared Spectroscopy (FT-IR) is a type of vibrational spectroscopy. Fourier Transform Infrared (FT-IR) spectroscopy is a chemical analytical method that utilizes the mathematical Fourier transform method to measure the wave number versus infrared intensity of light. It has been established that infrared rays are absorbed by the molecular vibrations. In the context of mathematical Fourier transform spectroscopy, the radiation intensity is considered as a function of time. Spectra of both high and rapid resolution can be obtained without the necessity of scanning each length separately. This method is used in molecular bond characterization. The structural characteristics of organic compounds, encompassing their functional groups, can be determined in a solid, liquid, gas, or solution states. The method facilitates the determination of bond types binding sites, and classification of the structure as aromatic or aliphatic. Fourier transform infrared spectroscopy (FT-IR) is a material characterization technique extensively used in both research and industrial applications. The FT-IR 4700 boasts a spectral resolution of 0.4 cm^−1^ and a signal-to-noise ratio of over 35,000:1, rendering it optimal for high-resolution applications, such as gas analysis.

In the context of routine sample analyses, the FT-IR 4000 (FT-IR 4000, ATR-PRO ONE instrument, Hachioji, Tokyo, Japan) was used with a single bound ATR-PRO ONE accessory, high-throughput prisms such as monolithic diamond, germanium (ideal for carbon black samples) or ZnSe. Pressure clamps with a maximum capacity of 10,000 psi ensure optimal sample contact with the crystal.

## 3. Results and Discussion

**Figure 3:**
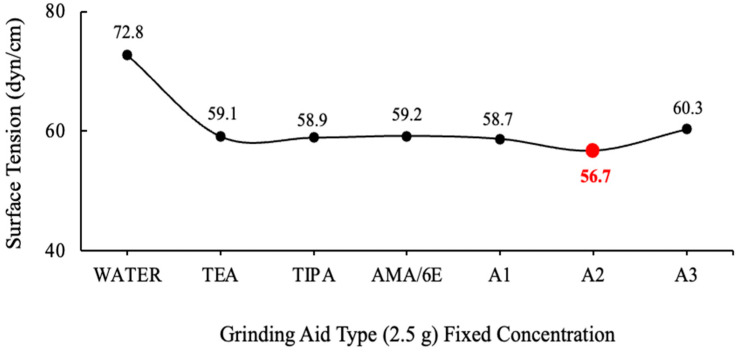
Surface tension data of fixed concentration (2.5 g) of the grinding aids.

**Figure 4:**
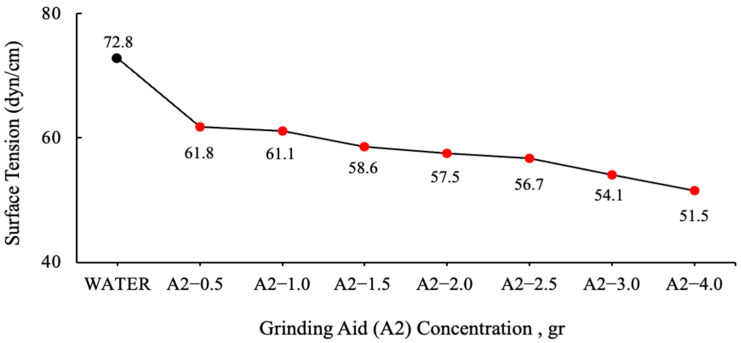
Surface tension data of varying A2 polymer-based grinding aid concentrations.

The graphs were generated by the calculating mean values. The values of grinding chemicals whose surface tensions were measured using the DuNouy Ring Method are displayed in Figure 3 and Figure 4.

The surface tension of water has been measured to be 72.8 dyn/cm at 20 °C. It is important to note that water necessitates a force of 72.8 dynes disrupt the intermolecular relationships that exist on the liquid surface for a distance of 1 cm [37]. It has been demonstrated that the solutions prepared with different ratios of A2 polymer require less force to break the tension between the liquid surface and the air surface relative to water.

**Figure 5:**
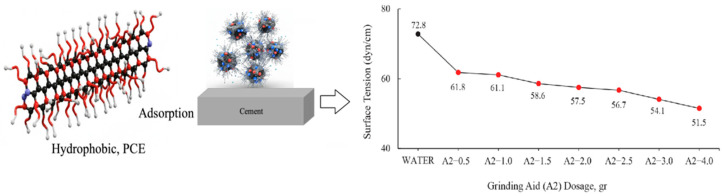
Relationship between PCE adsorption and surface tension of cement particles.

According to PCE dosages, the general working mechanism of cement particles with respect to surface tension is given in Figure 5.

Surface tension is defined as a property of a liquid surface that causes it to act as if it were a stretched elastic membrane [38]. The surface tension of the air–liquid interface is attributable to the imbalanced molecular cohesive forces exerted on the molecules of an interface [39]. The interfacial layer is defined as a quasi-three-dimensional physical region, with a thickness measuring approximately 10 nm. Concerning molecules situated beneath the surface of a liquid, the intermolecular forces exerted by these molecules can be considered to offset each other. Consequently, the molecules within the liquid are in a state of force equilibrium [40]. However, for molecules situated at the air–liquid interface, the intermolecular force exerted by the bulk molecules (i.e., cohesion caused by van der Waals force) is significantly stronger. This balance of forces gives rise to surface tension. As demonstrated, surface tension acts parallel to the interface [41].

**Figure 6:**
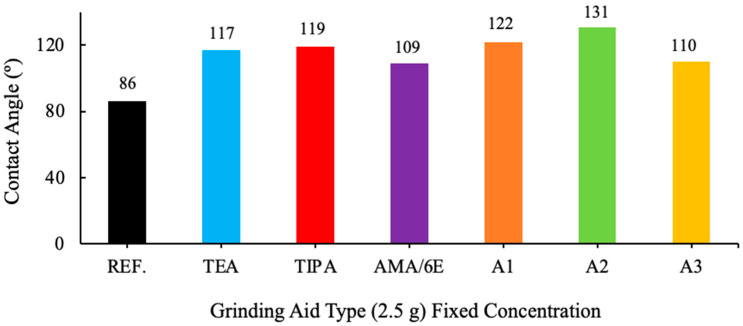
Effect of different grinding aid types with fixed concentration (2.5 g) on the contact angle.

**Figure 7:**
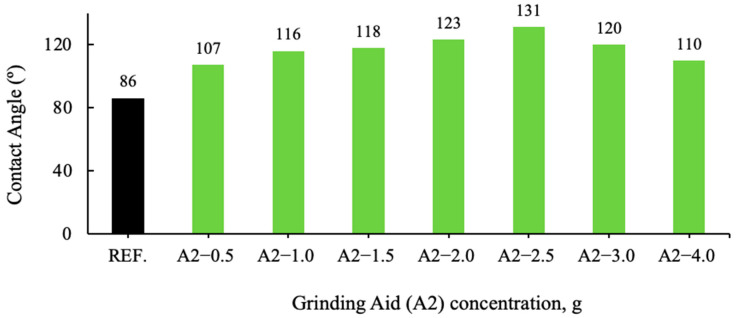
Effect of the varying A2 grinding aid concentration on the contact angle of cement samples.

The contact angles were measured with a goniometer on 13 samples prepared as pellets. The administration of four drops of pure water, measured out with precision and delivered via a syringe, was conducted for each pellet. The application was meticulously executed in a specific direction, namely from right to top to left to bottom. The values obtained from the computer for each drop were recorded automatically. Results were analyzed from two distinct perspectives: left and right. The mean values obtained are presented in Figure 6 and Figure 7.

An analysis of the contact angle data of various grinding additive cement types reveals a clear correlation with the degree of hydrophobicity (non-wetting properties). Cement types with a contact angle greater than 90° are designated as hydrophobic, while those with a contact angle less than 90° are classified as hydrophilic (wetting properties). Cement types with a contact angle greater than 140° are considered super hydrophobic, and those with a contact angle very close to 0° are defined as hydrophilic.

**Figure 8:**
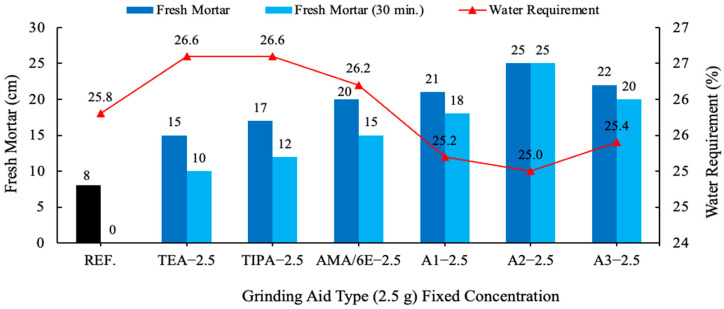
Effect of different grinding aid types with fixed concentration (2.5 g) on the slump flow.

**Figure 9:**
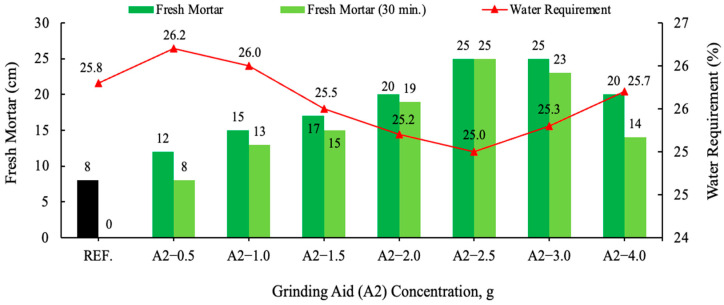
Effect of varying concentration of the A2 grinding aid on the slump flow.

The mortar test was conducted utilizing a mixture comprising 450 g of CEM I 42.5 R Portland Cement (PC), 225 g of water, 1350 g of sand, and first different types of grinding aids with 2.5 g and in a second step, a different amount of A2 grinding aid. The impact of time on the flowability of fresh mortars was examined, with samples analyzed both immediately after mixing and after a 30 min. waiting period. Workability represents a pivotal practical consideration within the domain of concrete technology. Consequently, the consistency of the material was subjected to a 30 min. durability test. The decline in consistency over time is concomitant with the progression of cement hydration. A consideration of the results for all mortars tested is warranted, as illustrated in Figure 8 and Figure 9.

**Figure 10:**
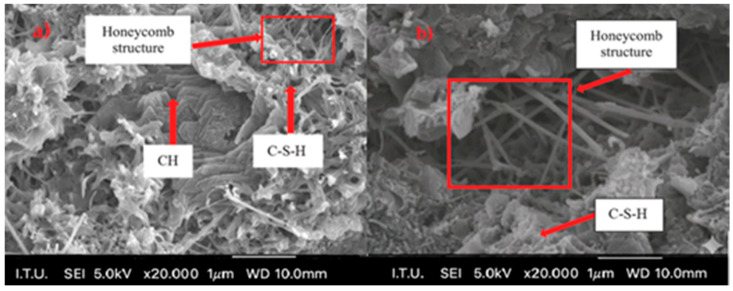
SEM images of the cement grains: (**a**) before, (**b**) after addition of superplasticizer.

In comparison with the result of the reference cement mortar devoid of grinding aid, all other cement mortars comprising various grinding aids yielded divergent outcomes. Fresh mortar tests performed with polycarboxylate-based A1–A2 and A3 grinding chemicals have been shown to exhibit the highest fluidity on the flow table when compared to other products. An analysis of the distribution of cement pastes on a spreading board after 30 min. reveals that the most effective result is achieved by the A2 grinding chemical, with a spread of 25 cm. Polymer grinding aids, especially A2 have the potential to be utilized as a chemical for the production of self-compacting concrete, a property attributable to their slump retention characteristics. Investigations have shown that polycarboxylate-based grinding aids support the homogeneous dispersion of cement grains, allowing them to move more easily past one another, thereby decreasing the agglomeration between cement particles. As illustrated in Figure 10, cement particles with and without a polymer grinding aid were shown.

The clinker used in both the reference and blended cement samples is identical. Therefore, numerical analysis was not performed at different hydration stages. Sulfate ions can react with the hydration products of cement to form expansive compounds such as ettringite (calcium aluminate sulfate hydrate) and gypsum (calcium sulfate). These reactions may lead to volumetric expansion of the concrete, causing cracking, reduced strength, and decreased impermeability. However, in our previously published study, while investigating the mechanical, physical, and surface chemistry properties of cement with amine-group-based polymer grinding aids, no volumetric expansion was observed. This finding supports further examination of the polymer’s mechanism [7,9].

**Figure 11:**
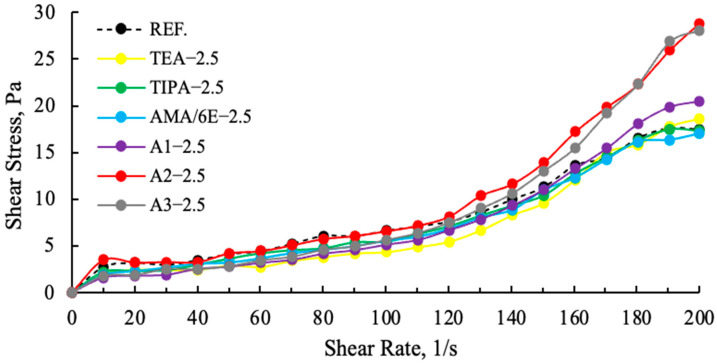
Effect of different grinding aid types with fixed concentration (2.5 g) on the shear stress.

**Figure 12:**
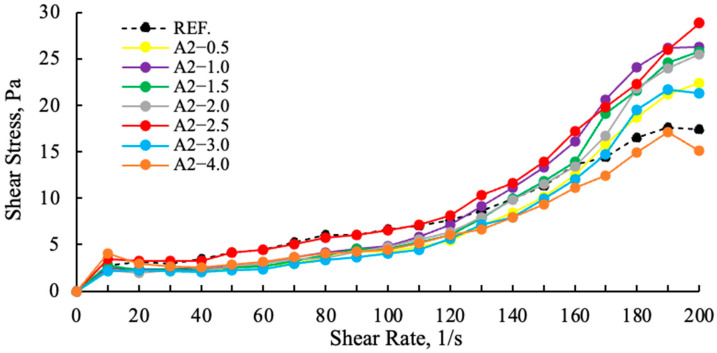
Effect of the A2 grinding aid concentration on the shear stress of cement samples.

Fundamental studies on the behavior of polymer-based chemical additives in their reactions with well-characterized cements have improved our understanding of the next generation of chemicals and paved the way to produce new optimized products [42]. Polymer admixtures consist of a main polymer backbone composed of carbon atoms to which carboxylic acid and long-chain polyether side groups are attached. The combination of a shorter polymer backbone and longer/more numerous ether side chains results in larger and longer lasting processability improvements [43]. As the concentration of superplasticizer increases, the yield stress decreases. At concentrations above a critical level, the flow transitions to a Newtonian state, where the interparticle attraction is suppressed by the additive [44,45]. As illustrated in Figure 11 and Figure 12, the shear stress values of cement samples ground with different grinding chemicals are indicative of this viscoelastic response.

As demonstrated in Figure 11, a comparison of cement samples containing amine group additives with those containing polymer group additives reveals higher slip ratios in the polymer-based samples. The A2—2.5 g shear stress is higher and more stable than the reference, other polymer and amine additives. This finding suggests that the viscosity values of polymer-based GAs are lower than those of amine group GAs, and that their water requirements are also lower [11]. In Figure 12, an examination of the effects of varying ratios on the slip ratios of A2 reveals that A2—2.5 g demonstrates a more stable increase, while A2—3 g and A2—4 g exhibit pronounced drops at 180 (1/s) as a result of tail-to-tail interaction. This situation lends support to the hypothesis that there will be rapid consistency losses in both cement and concrete.

**Figure 13:**
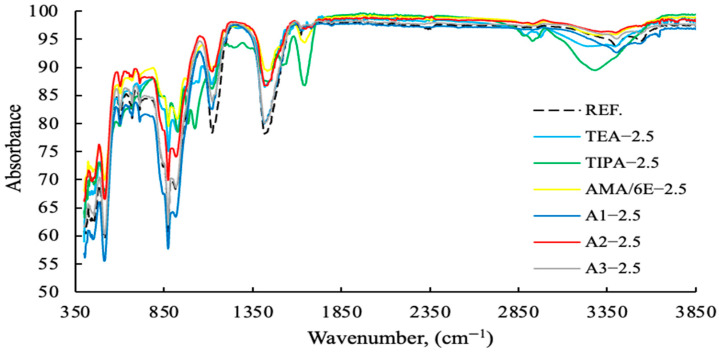
FT-IR data of cement samples with fixed concentration (2.5 g) with different grinding aid types.

**Figure 14:**
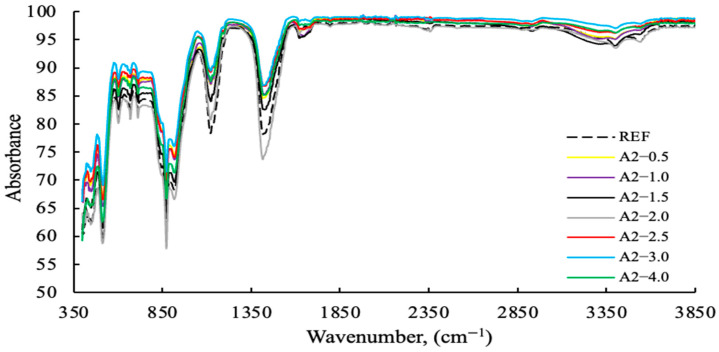
FT-IR data of cement samples with varying concentrations of the A2 grinding aid.

**Figure 15:**
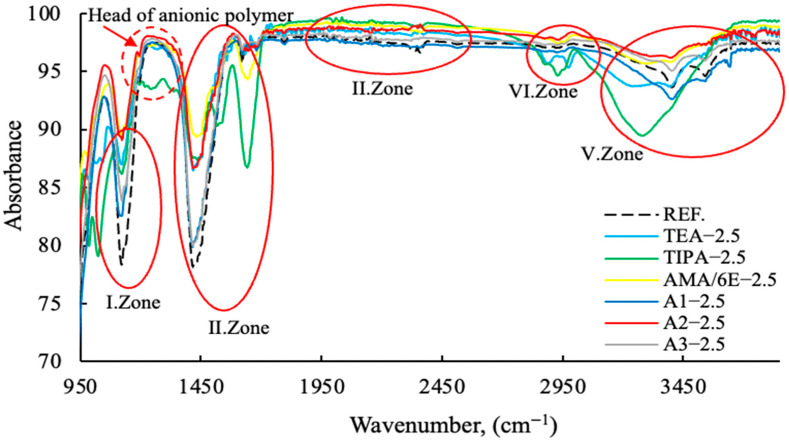
FT-IR data of cement samples with fixed concentration (2.5 g) different grinding aid types (Zone I-II-III-IV-V).

**Figure 16:**
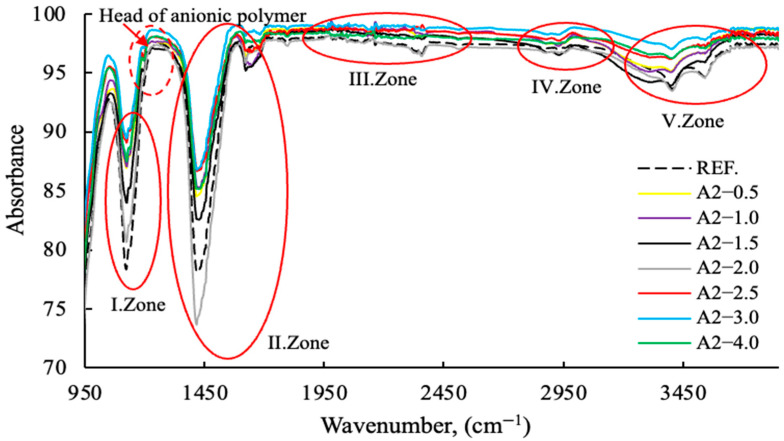
FT-IR data of cement samples with varying concentrations of the A2 grinding aid (Zone I-II-III-IV-V).

The FT-IR results with varying PCE (2.5 g fixed) and A2 grinding aids dosages from 0–4 g are shown in Figure 13, Figure 14, Figure 15 and Figure 16. The characteristic peaks can be approximately categorized into five regions.

As demonstrated in Figure 13, polymer-based GAs exhibit higher levels of absorption compared to amine-based GAs. As demonstrated in Figure 14, polymer-based GA A2—3 g exhibits notable efficacy as a reference and within its designated usage range.

As illustrated in Figure 15 and Figure 16, the anionic/cationic behaviors of polymers and amine groups in GA exhibit region-dependent variations, indicating that the mechanisms of action differ. This is consistent with the zeta potential data in the publication referenced [11], made by the same research team. Polymer-based grinding aids have a decisive effect on the grinding process of the head sections within the 1150–1350 wavenumber range.

The mechanisms of action of regional grinding aids are provided in Table 4.

The Fourier-Transform Infrared (FT-IR) spectroscopic data clearly delineates the component-specific structural impact of the A2—2.5 modification, primarily targeting the Si and O containing domains. The distinct intensification of the asymmetric stretching modes within the 950 to 1250 cm^−1^ region (I. and II. Zones), characteristic of Si-O-Si or Si-O-H moieties, signifies a pronounced enrichment or enhanced cross-linking of the silica network compared to the reference. Furthermore, the substantial profile variation observed in the O-H stretching envelope (V. Zone, ∼3000 to 3500 cm^−1^) confirms that the A2 component actively perturbs the hydrogen bonding equilibrium and the accessibility of internal hydroxyl groups. Conversely, the relative invariance of the C-H stretching bands (∼2850 to 3000 cm^−1^) suggests that the modification mechanism is directed towards the inorganic or polar components.

## 4. Conclusions

This research dealt with optimizing the cement rheology and hydrophobicity using polycarboxylate ether (PCE)-based grinding aids. The experimental findings revealed that the incorporation of polymer-based grinding aids significantly influenced the physicochemical properties of Portland cement. The reduction in surface tension observed with all additives, particularly for the A2 polymer, facilitated the nucleation and stabilization of air bubbles, thereby promoting cement hydration and dispersion [24,38,41].

The contact angle measurements further demonstrated that the A2 polymer exhibited a considerably higher value (131.7°) compared to other additives. This indicates a stronger hydrophobic character, suggesting that A2 enhances the interaction between cement particles and contributes to improved surface modification.

Rheological analyses showed that the A2 additive provided the most stable performance, maintaining a 25 cm slump flow value consistently after 30 min. The shear stress behavior indicated that A2 promoted rapid structural development within the first seconds, followed by stabilization, which highlights its effectiveness in controlling flow behavior and setting kinetics of cement suspensions. The investigation was conducted into the shear stress values of cement samples obtained using different grinding chemicals at shear rate of 200 (1/s). It was determined that the reference cement had a value of 17.45 Pa, while A2—2.5 g had a value of 28.82 Pa. During the same shear rate, the viscosity values of the reference cement were 67.22 cP, while the viscosity value of A2—2.5 g decreased to 55.31 cP (for about 20%).

FT-IR analysis confirmed the chemical interactions between the grinding aids and cement components across different spectral regions. In particular, stronger CH_3_ bonding features were observed in samples with A2, while the lowest water demand was recorded in A2-modified cement suspensions [46,47,48,49,50]. These findings collectively demonstrate that A2 outperforms other grinding aids in enhancing surface properties, reducing water requirement, and improving the overall rheological stability of cement systems.

The results of experiments demonstrated that chemical A2 exhibited higher levels of activity in terms of dispersant and agglomeration-preventing surface activity when compared to the other chemicals. Overall, the A2 polymer displayed superior dispersing and anti-agglomeration performance compared with other additives.

Rheological measurements demonstrated a decline in plastic viscosity with increasing utilization of polymer-based grinding chemicals. At the optimum dosage concentration, the flow transitioned to a near Newtonian flow state by reducing the attraction between the additive and the cement particles [47,50]. This phenomenon is attributed to the enhanced repulsive forces between cement particles, a reduction in agglomeration, and the preservation of a homogeneous structure within the system. The rheological results, with 2–2.5 g of polymer-based grinding chemicals, also corroborate this claim [11].

In comparison with conventional amine-group grinding chemicals, the utilization of the synthesized A2 polymer at the optimal concentration (2.5 g) offers environmental benefits by reducing both polymer and grinding energy costs, as well as increasing compressive strength by approximately 10%.

In a recent study by the same research team, zeta potential measurements confirmed the superior performance of the polymer-based A2 additive [11]. The A2-modified cement exhibited a zeta potential of −8.98 mV, indicating a stronger negative repulsive force compared to amine-based grinding aids. This enhanced repulsion improved particle dispersion, promoted a more micro cement structure and contributed to the higher compressive strength of the hydrophobic cement product.

Consequently, as A2 is formulated as a polymer-based grinding chemical, it can function as an aid in concrete chemicals due to its high slump behavior and slump retention in self-compacting concrete designs when utilized in concrete production as a concrete chemical, while rendering the system hydrophobic through the silanols it contains.

## Figures and Tables

**Table 1:** The chemical composition of the clinker.

Oxides	SiO_2_	Al_2_O_3_	Fe_2_O_3_	CaO	MgO	SO_3_	LOI	Na_2_O	K_2_O	Cr_2_O_3_	Mn_3_O_4_
**Wt (%)**	20.55	4.71	3.55	66.63	1.58	1.28	0.44	0.12	0.91	0.04	0.19

**Table 2:** The physical and chemical properties of the grinding aids.

Polymer	TEA	TIPA	AMA/6E	A1	A2	A3
**Solid rate (%)**	88	78	52	50	50	50
**pH**	11.60	7.80	5.60	5.80	5.73	6.00
**Color**	Transparent	Transparent	Brown	Light Yellow	Transparent	Transparent
**Density(g/cm^3^)**	1.12	1.02	1.07	1.05–1.15	1.05–1.15	1.05–1.15
**Molecular W. (g/mol)**	149.19	191.27	58,700	59,000	60,000	58,900

**Table 3:** Fixed ratio of different grinding chemicals and various contents of A2 solutions for 100 mL total volume.

Grinding Aid Type and Concentration	Various Types of Grinding aid Recipes for a 100mL Solution
Reference	100 g water
TEA—2.5 g	12.5 TEA g + 87.5 g water
TIPA—2.5 g	12.5 TIPA g + 87.5 g water
AMA/6E—2.5 g	12.5 AMA/6E g + 87.5 g water
A1—2.5 g	12.5 A1 g + 87.5 g water
A2—0.5 g	2.5 A2 g + 97.5 g water
A2—1.0 g	5.0 A2 g + 95.0 g water
A2—1.5 g	7.5 A2 g + 92.5 g water
A2—2.0 g	10.0 A2 g + 90.0 g water
A2—2.5 g	12.5 A2 g + 87.5 g water
A2—3.0 g	15.0 A2 g + 85.0 g water
A2—4.0 g	20.0 A2 g + 80.0 g water
A3—2.5 g	12.5 A3 g + 87.5 g water

**Table 4:** Interpretation table of FT–IR Wavenumber (cm^−1^) in Figure 13, Figure 14, Figure 15 and Figure 16.

Wavenumber (cm^−1^)	Interpretation
950	Attributed to silicate phases; associated with Si–O stretching vibrations [45].
950–1100	A2 is a polymer-based grinding chemical which demonstrates the highest wavelength dispersion at 2.5 g. In this region, TEA-TIPA and Ref. exhibit the lowest wavelength dispersion [46,47].
1100	Assigned to sulfate phases; corresponds to S–O stretching vibrations [45,47].
1150–1250	Head of anionic polymer group [48].
1150–1350	This is the point at which the heads of the chemicals interact. The finding that A2—2.5 g polymer-based grinding chemicals exhibit a higher wavelength than the amine group grinding chemicals and the reference cement serves to substantiate the dispersion effect of polymers on the grinding process [48].
1380	Surfactant tail group; CH_3_–R bending vibration [49].
1420	Surfactant tail group; CH_2_ bending vibration [49].
1468	Surfactant tail group; CH_2_ bending vibration [46].
1380−1580 (broad band)	Region characteristic of surfactant head groups and tail modes; in cationic or nonionic surfactants, a broad absorption feature is typically observed [49].
1950-2450	Surfactant chain; strong Si−OH bending. Silanols have been demonstrated to render the system either hydrophobic or hydrophilic [50].
2850	Surfactant alkyl chain; distinct CH_3_ stretching vibration [47].
2870	Surfactant alkyl chain; weak CH_3_ stretching vibration [47].
2920	Surfactant alkyl chain; distinct CH_3_ stretching vibration [47].
2955	Surfactant alkyl chain; weak CH_3_ stretching vibration [47].
2900−3500	This phenomenon is associated with the retention of water molecules between the molecules of a substance. In this region, A2—2.5 g polymer-based grinding chemicals have demonstrated a reduced water demand in comparison to amine groups [47].
3200−3450	Water-associated region; reflects structural and chemical changes of water molecules adsorbed onto the cement particle surface [47].

## Data Availability

The original contributions presented in this study are included in the article. Further inquiries can be directed to the corresponding author.

## References

[1] Ferrari L., Kaufmann J., Winnefeld F., Plank J. (2012). Multi–method approach to study influence of superplasticisers on cement suspensions. Cem. Concr. Res..

[2] Plank J., Zhimin D., Keller H., Hössle F.V., Seidl W. (2010). Fundamental mechanisms for polycarboxylate intercalation into C_3_A hydrate phases and the role of sulfate present in cement. Cem. Concr. Res..

[3] Palacious M., Puertas F. (2005). Effect of superplasticiser and shrinkage reducing admixtures on alkali–activated slag pastes and mortars. Cem. Concr. Res..

[4] Lei L., Plank J. (2014). A study on the impact of different clay minerals on the dispersing force of conventional and modified vinyl ether based polycarboxylate superplasticizers. Cem. Concr. Res..

[5] Ng S., Plank J. (2012). Interaction mechanisms between Na montmorillonite clay and MPEG-based polycarboxylate superplasticizers. Cem. Concr. Res..

[6] Lei L., Plank J. (2012). A concept for a polycarboxylate superplasticizer possessing enhanced clay tolerance. Cem. Concr. Res..

[7] Dengiz Özcan E., Çinku K., Özdamar Ş., Ergin H., Özkan Ş.G. (2021). Investigation of the effect of polymer–based novel grinding aids on cement grinding efficiency. J. Appl. Polym. Sci..

[8] Çinku K., Akkaya U.G. (2024). Study of hydrophobic cemented paste backfill (H–CPB) to prevent sulphate attack. Heliyon.

[9] Akkaya U.G., Çinku K., Yılmaz E. (2021). Characterization of Strength and Quality of Cemented Mine Backfill Made up of Lead–Zinc Processing Tailings. Front. Mater..

[10] Martins J.R., Rocha J.C., Novais R.M., Labrincha J.A., Hotza D., Senff L. (2024). Zeta potential in cementitious systems: A comprehensive overview of influencing factors and implications on material properties. J. Build. Eng..

[11] Çinku K., Dengiz Özcan E., Özdamar Ş., Ergin H. (2025). Investigation of the Effects of Polymer–Based Grinding Aids on the Surface Chemistry Properties of Cement. Polymers.

[12] Liu J., Wang K., Zhang Q., Han F., Sha J. (2017). Influence of superplasticizer dosage on the viscosity of cement paste with low water-binder ratio. Constr. Build. Mater..

[13] Gowthami D., Sharma R.K. (2023). Influence of Hydrophilic and Hydrophobic modification of the porous matrix on the thermal performance of form stable phase change materials: A review. Renew. Sustain. Energy Rev..

[14] Zeng X., Lan X., Zhu H., Long G., Xie Y. (2023). Using stirring power curves to investigate the air–entrainment and mechanical properties of cement mortar at low air pressure. Constr. Build. Mater..

[15] Shi Y., Wang Z., Li Z., Li Y., Liu J., Zhu Y. (2018). Effect of atmospheric pressure on performance of AEA and air entraining concrete. Adv. Mater. Sci. Eng..

[16] Li Y., Wang M., Liu S., Zhang Y., Lu N., Zhou X., Zeng X. (2019). The influence of atmospheric pressure on air content and pore structure of air–entrained concrete. J. Wuhan Univ. Technol. -Mater. Sci. Ed..

[17] Zhou Y., Sun W., Ling Z., Fang X., Zhang Z. (2017). Hydrophilic modification of expanded graphite to prepare a high–performance composite phase change block containing a hydrate salt. Ind. Eng. Chem. Res..

[18] Kou Y., Liu Q., Zhang H., Wang X., Liu J., Xie W. (2021). An intrinsically flexible phase change film for wearable thermal managements. Energy Storage Mater..

[19] Behzadian R., Shahrajabian H. (2019). Experimental study of the effect of nano–silica on the mechanical properties of concrete/PET composites. KSCE J. Civ. Eng..

[20] Prziwara P., Breitung-Faes S., Kwade A. (2018). Impact of grinding aids on dry grinding performance, bulk properties and surface energy. Adv. Powder Technol..

[21] Altun O., Altun N., Torgal V.P., Benzer H. (2015). Utilization of grinding aids in dry horizontal stirred milling. Powder Technol..

[22] (2011). Cement. Composition, Specifications and Conformity Criteria for Common Cements.

[23] Katsioti M., Katsiotis N., Kalleris M., Moropoulou A. (2009). Characterization of various cement grinding aids and their impact on grindability and cement performance. Construct. Build. Mater..

[24] Chipakwe V., Lartey O., Yalley S., Konadu B. (2020). A critical review on the mechanisms of chemical additives used in grinding and their effects on the downstream processes. J. Mater. Res. Technol..

[25] Kobya V., Kaya Y., Akgümüş F.E., Kaya Y., Mardani N., Mardani A. (2025). Sustainable Cement Production: TEA-TIPA as Grinding Aids: Optimizing Ratios for Efficiency and Environmental Impact. Polymers.

[26] Wang Z., Ma Y., Zhang P., Zhao Z., Deng C. (2023). Effects of grinding aids on the grinding kinetics and surface morphological characterization of quartz. Adv. Powder Technol..

[27] Jia J., Wang Y. (2022). Interactive effects of admixtures on the compressive strength development of Portland cement mortars. Buildings.

[28] Kaya Y., Çinku K., Akkaya U.G. (2024). Effect of modified Triethanolamine on grinding efficiency and performance of cementitious materials. Talanta Open.

[29] Kaya Y., Kobya V., Samadpour N., Altun O., Ozcan A., Kaya Y., Mardani A. (2025). Evaluation of polycarboxylate ether-based grinding aids on clinker grinding performance: The influence of pH. J. Sustain. Cem.-Based Mater..

[30] Popov A., Hristova T., Stoyanova D., Hristova S., Mladenova S. (2024). Effect of grinding aids on cement properties and grinding process. J. Chem. Technol. Metall..

[31] Zhan P., Wang J., Yu W., Deng Z., She A., Zuo J., Li W., Xu J. (2025). Insights into the hydration kinetics, microstructure and early strength of Portland cement containing synthetic C-S-H/PCE nanocomposites. Cem. Concr. Com..

[32] Tafesse M., Kim H. (2019). The role of carbon nanotube on hydration kinetics and shrinkage of cement composite. Compos. B Eng..

[33] Papo A., Piani L. (2004). Effect of various superplasticisers on the rheological properties of Portland cement pastes. Cem. Concr. Res..

[34] Banfill P.F.G. (1981). A viscometric study of cement pastes including a note on experimental techniques. Mag. Concr. Res..

[35] Tattersall G.H., Banfill P.F. (1983). The Rheology of Fresh Concrete.

[36] Roussel N., Coussot P., Houthoofd B., Cizer Ö., De Schutter G. (2010). Steady state flow of cement suspensions: A micromechanical state of the art. Cem. Conc. Res..

[37] Chateau X., Ovarlez G., Trung K.L. (2008). Homogenization approach to the behaviour of suspensions of noncolloidal particles in yield stress fluids. J. Rheol..

[38] The Editors of Encyclopaedia Britannica Surface Tension. Encyclopaedia Britannica.

[39] Zeng X., Zhou X., Liu S., Wang T., Wang T., Li Y. (2020). A review on bubble stability in fresh concrete: Mechanisms and main factors. Materials.

[40] Israelachvili J.N. (2011). Intermolecular and Surface Forces.

[41] Marchand A., Weiden J.H., van der Meer D. (2011). Why is surface tension a force parallel to the interface?. Am. J. Phys..

[42] Lan X., Zhang J., Zhong Y., Zhang C., Wang J., Li Y. (2023). How nano-bubble water and nano-silica affect the air-voids characteristics and freeze-thaw resistance of air-entrained cementitious materials at low atmospheric pressure. J. Build. Eng..

[43] Zeng X., Zhang H., Zhou X., Wang T., Liu S., Wang T. (2021). Investigation on air–voids structure and compressive strength of concrete at low atmospheric pressure. Cem. Concr. Compos..

[44] Cao R., Zhang Q., Wang M., Gao C. (2020). Interpreting the early–age reaction process of alkali–activated slag by using combined embedded ultrasonic measurement, thermal analysis, XRD, FTIR and SEM. Compos. B Eng..

[45] Kogelheide F., Kartaschew K., Strack M., Baldus S., Metzler-Nolte N., Havenith M., Awakowicz P., Stapelmann K., Lackmann J.-W. (2016). FTIR spectroscopy of cysteine as a ready–to–use method for the investigation of plasma-induced chemical modifications of macromolecules. J. Phys. D Appl. Phys..

[46] Scheuing D.R. (1991). Fourier Transform Infrared Spectroscopy in Colloid and Interface Science.

[47] Liu Q., Chen Y., Zhang T., Wang H., Deng X. (2020). Study of the air–entraining behavior based on the interactions between cement particles and selected cationic, anionic and nonionic surfactants. Materials.

[48] Cyr M., Legrand C., Mouret M. (2000). Study of the shear thickening effect of superplasticizers on the rheological behavior of cement pastes containing or not mineral additives. Cem. Conc. Res..

[49] Lewis J.A., Young J.F., Li S. (2000). Polyelectrolyte effects on the rheological properties of concentrated cement suspensions. J. Amer. Ceram. Soc..

[50] Qi L., Zhang T., Liu Q., Lv Y., Chen J. (2021). Model of the charged mosaic surface of the cement particle based on the adsorption behavior of surfactants using ATR–FTIR spectroscopy. Compos. Part B.

